# Gender sensitivity in career mentoring – a project report from the Medical Faculty of Leipzig University

**DOI:** 10.3205/zma001317

**Published:** 2020-03-16

**Authors:** Pauline Gaida, Sandy Kujumdshiev, Katarina Stengler

**Affiliations:** 1 Nervenzentrum Leipzig, Praxis für Neurologie, Psychiatrie und Psychotherapie, Leipzig, Germany; 2 Universität Leipzig, Medizinische Fakultät, Medizindidaktisches Zentrum, Leipzig, Germany; 3 Helios Park-Klinikum Leipzig, Klinik für Psychiatrie, Psychosomatik und Psychotherapie, Akademisches Lehrkrankenhaus der Universität Leipzig, Leipzig, Germany

**Keywords:** medicine, medical education, medical school, mentoring, gender, diversity, elective, gender sensitivity, career

## Abstract

**Objective: **The elective subject “career management for medical students” is presented as an example of teaching gender sensitivity issues among medical studies at Leipzig University. The project report shows the interim results of promoting gender-sensitive teaching at the Medical Faculty of Leipzig University, as well as the elective’s contribution to the development of gender sensitivity at the entire university.

**Method: **Project Description and Results show the organization/procedure, participants and detailed contents of the elective since it began in Winter Term 2010/11. The research examines the elective’s mandate at the Medical Faculty and beyond, i.e. by comparing with the efforts of other universities.

**Results:** The elective is the first subject for credit within the clinical curriculum of medical studies at Leipzig University that connects the topics of gender sensitivity and career management. It creates a view of the specialties of medicine and research as they relate to gender, and also on the options of a medical career and touches the topic of gender equality. A faculty survey in the winter semester of 2011/12 reveals that nearly one third of the medical students want an extension of the curriculum around the topic of gender or even an independent subject “Gender Medicine”. The elective is part of a cycle promoting gender equality at Leipzig University.

**Conclusion: **The elective initiates and continues the implementation of gender-sensitive teaching at the Medical Faculty of Leipzig University. The management of the elective aims at the permanent establishment of the subject in the curriculum in order to encourage career ambitions early – especially for women.

## Zusammenfassung

**Zielsetzung: **Das Wahlfach „Karriereplanung für Medizinstudierende“ wird als Beispiel für die Lehre von geschlechtersensiblen Aspekten im Leipziger Medizinstudium vorgestellt. Welche Zwischenbilanz kann dabei für die Förderung der geschlechtersensiblen Lehre an der Medizinischen Fakultät der Universität Leipzig gezogen werden? Welchen Beitrag leistet das Wahlfach zur gesamtuniversitären Entwicklung einer Geschlechtersensibilität?

**Methodik: **In Projektbeschreibung und Ergebnisteil werden Organisation/ Ablauf, Teilnehmer*innen und Inhalte des Wahlfachs seit Start im Wintersemester 2010/11 vorgestellt. Die Forschungsfragen untersuchen, welchen Auftrag die Lehrveranstaltung auf Fakultätsebene und darüber hinaus erfüllt. Ein Instrument hierbei ist der Vergleich mit anderen Hochschulen.

**Ergebnisse:** Lokal stellt das Wahlfach als erste offizielle Studienleistung im klinischen Studienabschnitt den Zusammenhang zwischen den Themen „Geschlechtersensibilität“ und „Karriere“ her. Die Lehrveranstaltung bedient inhaltlich die Geschlechtersensibilität im Fachkontext Medizin und Forschung, die Orientierung im Karrierefeld Medizin und den Gleichstellungsdiskurs. In einer Fakultätsumfrage im WS 2011/12 wünschen knapp ein Drittel der Studierenden eine Angebotserweiterung – bis hin zum eigenständigen Fach "Gender Medizin". Das Wahlfach ist Bestandteil eines Kreislaufes zur Förderung des Gleichstellungsprozesses an der Universität Leipzig.

**Schlussfolgerung: **Die Implementierung einer geschlechtersensiblen Lehre an der Medizinischen Fakultät der Universität Leipzig wird durch das Wahlfach initiiert und fortgesetzt. Ziel der Wahlfachleitung ist die Etablierung einer curricularen Lehrveranstaltung dieser Art zur frühzeitigen Förderung der – insbesondere weiblichen – Karriere.

## 1. Introduction

In their 2013 equality report, Leipzig University (LU) conjectured “a particular problem of compatibility within the university medicine” [1] and broke it into four dimensions: clinic, research, teaching and personal life. Earlier in 2008, the Saxon comprehensive university committed itself to implement the German Research Foundation’s “research-oriented standards on gender equality”. The LU consolidated the gender equality process by setting up the university-specific development plan (2011) and the equality policy (2013) [2], [3]. On that basis, the LU successfully applied for the “Women Professors Programme II” grant from the Federal Ministry of Education and Research (BMBF) [4]. The Medical Faculty (MF) of the LU considerably contributed to the equality policy through its mentoring program for female physicians working on habilitation (“MentHaProf”). This project, supervised by Prof. Katarina Stengler, who was the equal opportunity commissioner of Leipzig University Medicine (LUM) at the time, was a significant step in order to counteract the current state: “medicine is female – at the base, not at the top”. At the same time, the MF of the LU increased the focus on promoting women’s academic careers early: They expanded the elective “career management for medical students”, which had started three years earlier. This subject, which has now been established for ten years, considers the four dimensions of human medicine mentioned above as they relate to gender. The Project Description presents the legal framework and the learning theory assumptions of the elective subject subdivided into organization/procedure, participants and contents. The Results are a summary report about the perennial conduct. Finally, the discussion contextualizes the elective by means of the following questions:

1. What are the interim results for the promotion of gender-sensitive teaching at the MF of LU – as they relate to the establishment of the elective “career management for medical students”?
2. Is it legitimate to interpret the establishment of the elective “career management for medical students” at the MF of LU as a contribution to improving gender sensitivity for the entire university?

## 2. Project Description

### Organization/procedure

The elective subject has been offered to medical students since Winter Term 2010/11 by the MF of the LU. It was approved by the local study committee and faculty council. It is one of many electives for the official course distribution requirements, which need to be fulfilled by every student of advanced medical studies to get admitted for second state examination [5]. Regarding this the Saxon state audit office introduced the topic “gender aspects in medicine” as an amendment to their Regulation of Medical Approbation [https://www.gesetze-im-internet.de/_appro_2002/BJNR240500002.html]. The regulations require that at most six medical students are entrusted to a single instructor or mentor. The elective is designed as a one-week course, consisting of 27 lessons of 45 minutes each. The students’ work and contributions throughout the course are graded by the supervising instructor at the end of the week. An evaluation of the class can be provided by faculty if the instructor requests it. Several times during the elective, two groups of six students and at least two instructors congress to discuss in plenum. The subject is matter of the educational branch of the Institute for Social Medicine, Occupational Health and Public Health of the LU. Some lessons take place in the mentor’s occupational areas. 

#### Participants

The elective is presumed to be a short-term mentorship. Medical students are mentees. They can register for the course in their ninth semester. The mentors are recruited by the managing instructor from the diverse field of medicine. In this way the mentees meet with several professors, chief physicians, general practitioners and other office-working physicians, researchers, interns, and also with service providers like coaching experts or management consultants. These experts play a key role since they teach the mentees by personal interactions.

#### Contents

The key line of the LU equality report which points out “a particular problem of compatibility within the university medicine” [1] provides a general introduction for the one-week elective to the students. Being aware of this challenge becomes the common starting point for the week: How to create a constant balance of the four dimensions of a physician’s life – clinic, research, teaching and personal life? The interactions between mentors and mentees use flash mentoring [https://www.insala.com/Articles/what-is-flash-mentoring.asp]. Supportive questions are: Do you want to start out as an intern in a hospital? Would you prefer a general public hospital to a university clinic? Do you care for any additional conditions? Career management based on economic maximum principle brings focus to the benefit of synergy and claims calculability. The mentorship inspires the students to shape their own career. Individual expectations of the students from the subject are welcome with willingness to respond and fulfill. While the mentors keep up their main assignment as role models, they bear in mind the opportunities for bidirectional learning through first-hand interaction [6]. The course is a stage for mentors with stereotypical occupations. Traditionally antagonistic occupations like researcher versus practitioner host discussions with the students. Anticipated differences of opinion are the topic, e.g. work setting differences like hospital versus medical practice. Mentors and mentees talk about work time models and job-family compatibility and compare medical practice with medicine-related occupations. During the lecture the students are taught basic knowledge about equal opportunity, gender equality, career strategy, leadership qualities, networking and scholarships, as well as time- and self-management in order to promote resilience. The goal of the subject is to help medical students enjoy evolving and visualizing their own career ambitions through short-term mentorship, keeping in mind the LU’s goal of “actual equality between women and men“ [7].

## 3. Results

### Organization/procedure

**Figure 1:**
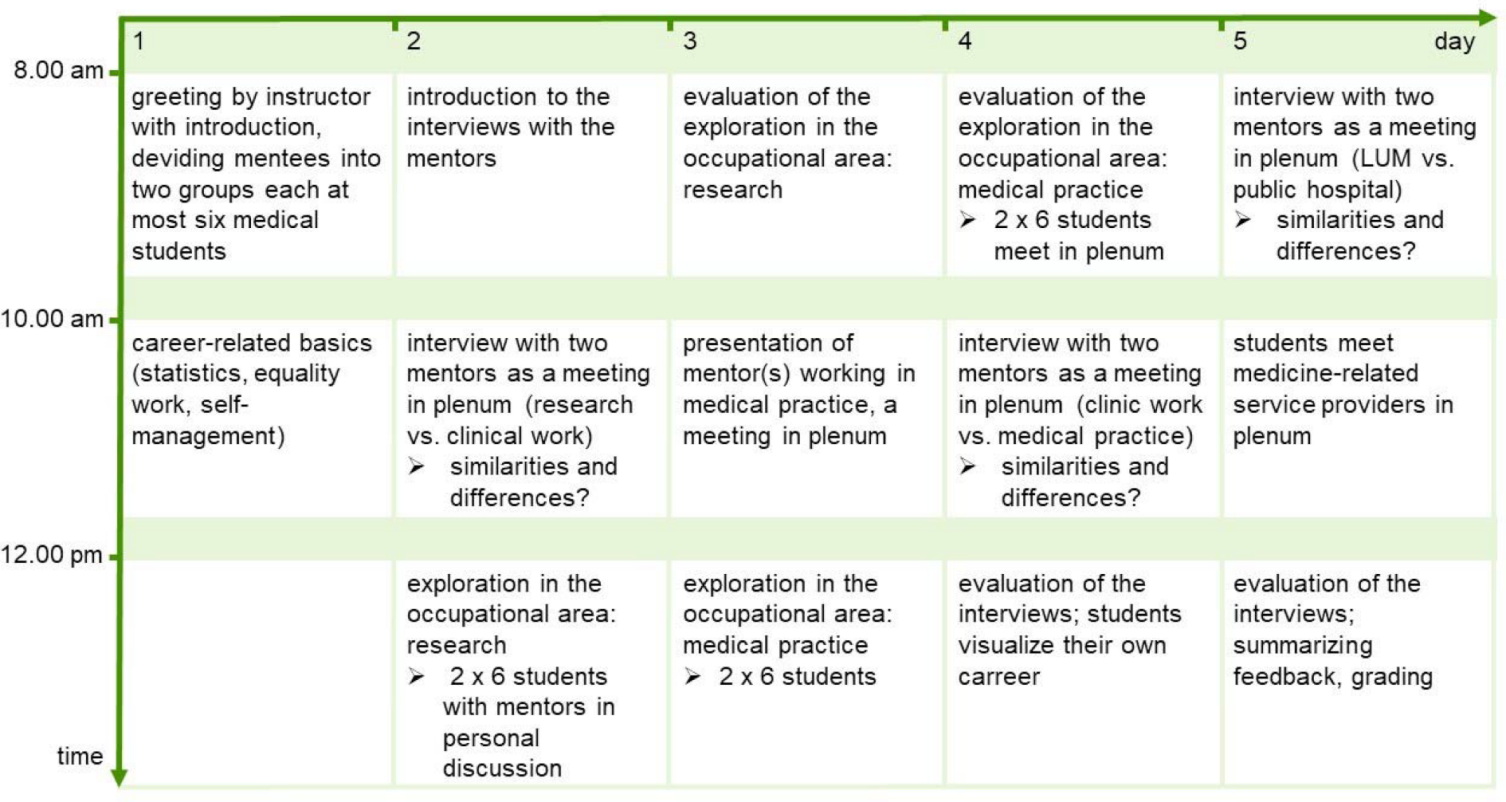
Schematic schedule of the elective “career management for medical students” at the Medical Faculty of Leipzig University

Since its implementation in 2010, the elective always took place in the winter semester. Figure 1 (Fig. 1) shows a schematic schedule. The emphasis of the course was on mentoring with 15 classes of 45 minutes each. Further sessions were used for theory and evaluation. There was always a ratio of at least one mentor for six students. If necessary, the managing instructor or the teaching assistant took on the role of a second mentor. The elective helped its participants to reflect on role-models and role-identification more consciously. The elective was evaluated through daily feedback between course instructor and students as well as by summarizing feedback at the end of the course and also by intervision of managing instructor and mentors. There has not been a written evaluation from the students.

#### Participants

To date, 108 medical students participated in the elective. In the first years of the elective, up to 15 students could enroll for the course. As of 2015, up to twelve students were allowed to sign up. The gender ratio of the participating students was not recorded. Women and men were nearly equally represented among mentors. The feedback of all participants was solely positive, emphasizing the need to extend this teaching offer. The medical students stated increased knowledge about career management in general and gender equality in particular. The one recurring statement – especially from female students – was: “I thought of it as almost impossible to balance career and family. The common clichés should be questioned more frequently”. In discourse real framework conditions of certain professional fields became clearer and prejudices were weakened. All participants appreciated the interpersonal exchange. The mentors emphasized the importance of fundamentally reconsidering professional stereotypes – such as surgery is a presumed male domain. Still these students’ introjections would very likely have a lasting effect on career planning, even thought professional conditions have already changed.

**Figure 2:**
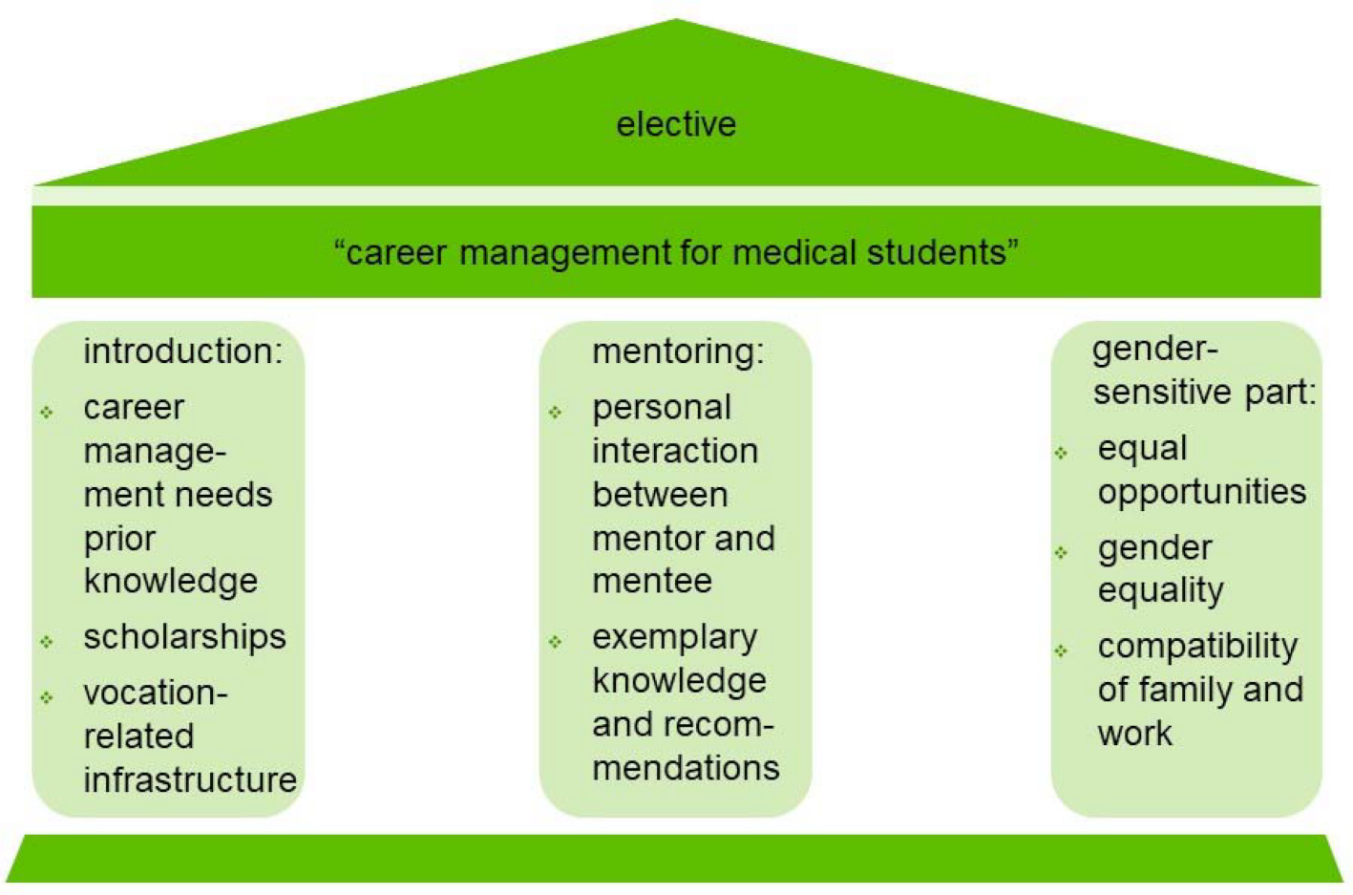
Contents of the elective “career management for medical students” at the Medical Faculty of Leipzig University

Contents: The contents of the elective consists of three parts (see figure 2 (Fig. 2)).

For the most part, these contents were taught interdependently:

1. The multilayer character of the five-day intensive program was explained to the medical students in the opening lecture. It illustrated that “career management for medical students” is more than just considering what medical specialty to enter into, and that for any medical discipline the gender-sensitive point of view and treatment is becoming the gold standard [8]. Students were told that career management requires solid prior knowledge about the benchmarks that can be found in human medicine and how professional prioritization can happen.
2. In the mentoring-based part, the most important course content was the personal interaction between mentor and student. The course instructor explicitly called out a confidentiality agreement. This created a protected space for asking individual questions. In conversations about their work and biography with the students, mentors were able to convey exemplary knowledge and recommendations to them. The mentors also guided explorations of their occupational fields. The specific teaching assignment was to promote one’s own awareness of the perception of roles: The medical students were told that personal career management is closely linked to one’s self-concept. Studying human medicine is the natural entry to a working life as a physician. But at the same time, it opens up a multitude of independent or overlapping professions. Particularly the work at a university hospital requires a physician to take on the triple role of maximum treatment, research and teaching. The students got to know different representatives of the LUM. In the elective, the debates and participants reflected the diversity of human medicine. Information about funding programs or vocation-related infrastructure emphasized the synergy.
3. The gender sensitivity part dealt with the topics of equal opportunities and the equality of men and women. The target group of the initial elective was solely female medical students – based on the idea of inspiring more female students to establish a career in medicine. Subsequently, the course was opened for male students as they also demanded career management as a course of study. Henceforth, at least one male student participated in the elective each time. The course catalyzed on distinct levels the ideational realization of the gender equality process that takes place everywhere: the participants discussed the professional role of women in medicine and the phenomenon of vertical segregation. This knowledge formed the basis for understanding gender equality issues. Two important questions were asked repeatedly to the mentors: “Have you ever felt sexually discriminated against during your career? Do you have a family, and does it compete with your profession?” The involvement of the former and recent equal opportunity commissioners of the LU, Mrs. Dr. Monika Benedix and Mr. Georg Teichert, was especially useful support. The medical students’ isolated view on gender sensitivity in pathology, diagnosis and therapy faded. They increasingly looked at an overall picture which comprises all the real people in the major and side areas of medicine: professionals as well as patients – with research given a high priority.

## 4. Discussion

### 4.1. What are the interim results for the promotion of gender-sensitive teaching at the MF of LU – as they relate to the establishment of the elective “career management for medical students”?

In Winter Term 2011/12, the MF of the UL surveyed its students and lecturers on behalf of the equal opportunity commissioner about the presence of gender sensitivity contents in medical studies. The survey's response rate was about 20% for the students (n=306; f=71.8%, m=28.2%) and about 38.8% for the lecturers (n=26; f=16,4%; m=83,6%). The results presented the current status, or rather the desire for change. 76 % of the lecturers emphasized the relevance of these ideas for their subject, yet only 61.5% confirmed implementing them. Merely 27.9% of the students were able to identify gender sensitive topics in their studies, for example due to the elective. The majority wants an extension of the curriculum, or even an independent subject “Gender Medicine”. Five years later, the conference series “Gender Perspectives in medicine (GPmed)” hosted by LU reiterated the demand for gender sensitivity in medicine: three inter-university events in 2016/ 2017 supported by the BMBF encouraged the exchange of experiences. The elective was presented as an optional course at the MF of the LU [9]. Nobody introduced any sort of curricular subject on gender medicine or gender sensitivity for the MF. A search of the “Study regulations for the course of study human medicine at Leipzig University” supports that impression: You cannot find a course or lecture de facto about gender medicine. The words sex or gender cannot be found in the list of subjects taught in pre-clinical and clinical study section [5]. Do some subjects incorporate gender sensitivity contents so they do not show up in the LU’s study regulation? The Charité – University Medicine Berlin entirely revised its curriculum in this aspect by hiring a “change agent” who guided the systematic integration of these concepts into every existing study event [10]. No comparable efforts have been taken at the MF of the LU, even though the study regulation in particular may benefit. Its § 28 “accommodation for disadvantage” furthermore proclaims that a candidate receives the offer to take any exam in an altered format if he brings evidence of a long-term or permanent disability [5]. The “Crosscurricular statute for regulation of admission, studies and examination of the Humboldt University of Berlin” from 2013 creates this idea about accommodation for disadvantage (§ 109(1)): 

“Any student who is unable to undertake graded or non-graded work on a particular date, in certain time limits, at a designated location, in the scheduled form or in the intended way on grounds of disability or chronic disease, pregnancy, caring for and bringing up children younger than eleven years, nursing relatives with extensive need for care […] or any other valid reason obtains accommodation for the disadvantages.” 

The elective deals proactively with compatibility of study or rather occupation with family, among other things. In Winter Term 2012/13 the Ulm University was the first nationwide to implement an independent course called “Gender Medicine” as part of its Medical Faculty’s curriculum so that each of the medical students acquires the key qualification “gender competence”. Applying this knowledge to patients and defining intrapersonal and interpersonal roles through self-reflection are the explicit goals [11]. The elective pursues the same objective. But since its introduction in 2010, it kept its nonobligatory character. Thanks to guided reflection, the students experience a significant change of perspective – conventional medicine gains gender sensitivity. For this purpose, in 2014 the University of Düsseldorf introduced a teaching tool called “Gender Lens” [12]. In 2016 the MF of LU founded the Centre for Medical Didactics. One of its tasks is to ensure the longitudinal curriculum for research competencies. By gaining capability in scientific reasoning, the students are meant to apply this skill in diagnostic and therapeutic decision making [13]. In this regard, new curricular courses could teach medical students about gender medicine and raise awareness for gender sensitivity as a feature of quality in modern science. The interim results within the promotion of gender-sensitive teaching at the MF of LU point out that the elective is the first explicit study offer.

#### 4.2. Is it legitimate to interpret the establishment of the elective “career management for medical students” at the MF of LU as a contribution to improving gender sensitivity for the entire university?

**Figure 3:**
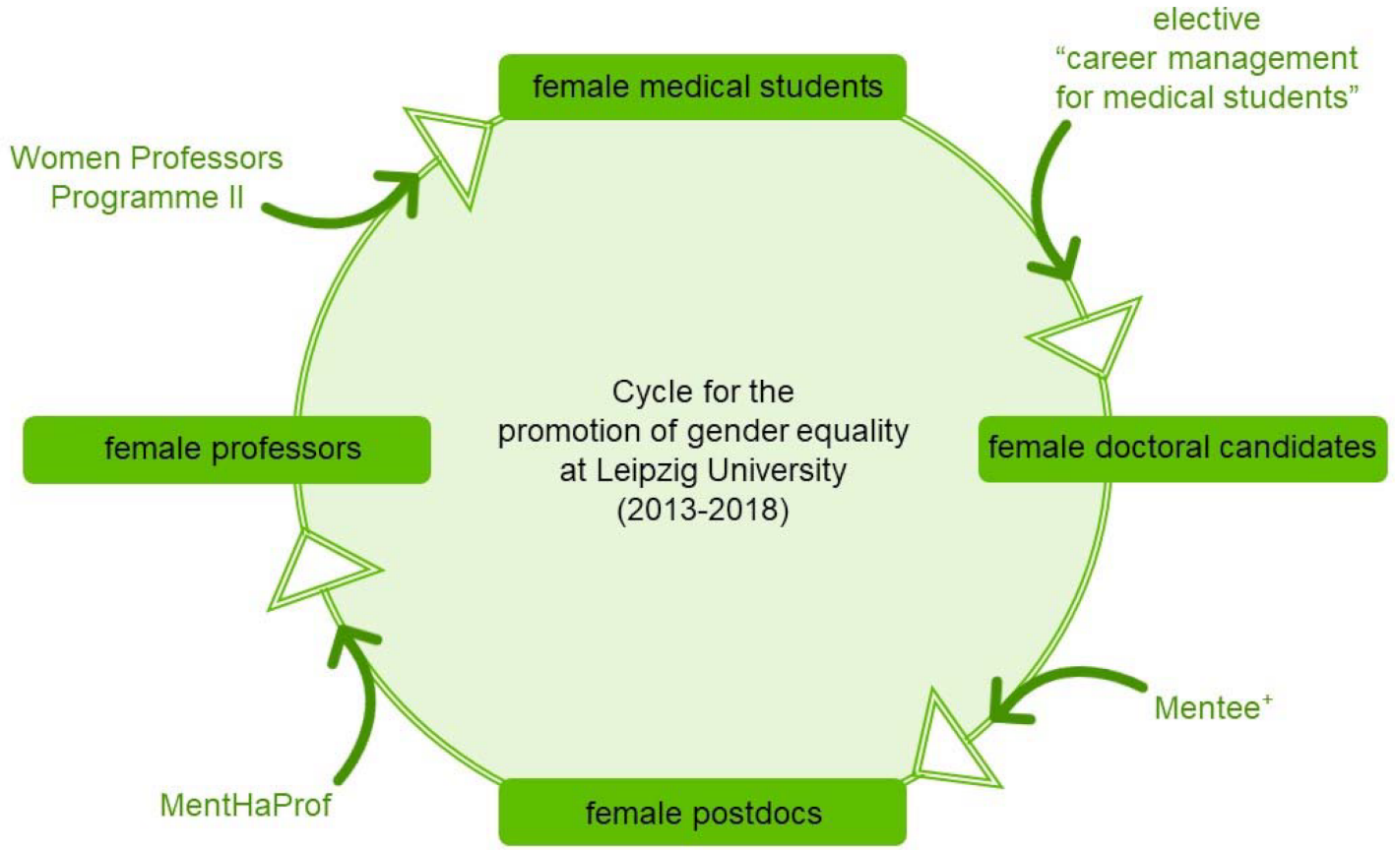
Cycle for the promotion of gender equality at Leipzig University

Yes. The small-group-based short-term mentoring program asks about all the individuals at the LU and the LUM, respectively. What are their experiences regarding their right to equal opportunities and equality? Political correctness should be evident in all actions. As a constructive tool in the elective the principle of error-friendliness by Urmila Goel is lived out: The irreversibility of the consequences of discrimination is set aside within the learning environment [14]. A simple example is the debate in the opening of the initial elective “career management for female medical students” for male students. After the first announcement of the seminar, male students expressed their sense of discrimination. Thereby, the elective became an example for the gender equity debate amongst the students. The Federal Anti-Discrimination Agency defines discrimination as “putting people to a disadvantage due to an attribute worthy of protection, such as gender”, meaning “equals are treated unequally” [15]. Why are only female students allowed to sign up for an official elective? The answer is provided by the second part of the definition: “Yet a disadvantage also exists when people with unequal preconditions are treated equally.” [16]. The elective aims to address that, because female physicians are affected in their career-management and -development by social, professional and family determinants in a way that they experience vertical careers less often than their male colleagues [17]. Due to its educational impact, the elective is supposed to contribute to an increased realization of female vertical careers. The dilemma about who gets admitted to the elective was resolved by opening the elective to every medical student – because actual gender equality requires the education of everyone. Andersson et al. emphasize this statement by proving that biological sex is a determinant for the awareness the topic of gender [18]. The KarMed analysis funded by BMBF and European Social Fund shows that medical students still think of the medical profession as a male-dominated occupation [19]. The elective deals with this idea. It makes evident that modern medicine provides models of female leadership at every level of the hierarchy. The elective was listed in the LU’s equality report (2013) in a large number of prestigious mentoring programs [2]. In this way the course played its part in the LU’s proposal for the funding of the “Women Professors Programme II”. The funding period stretched into 2018. That completed a diversely promoted cycle of education aiming at sustained gender equality at the LU (see figure 3 (Fig. 3)). Thus, the elective was able to contribute directly and indirectly to the development of gender sensitivity at the entire university.

## 5. Conclusion

What are the implications? In its guiding principles, the LU proclaims: “The university promotes actual equality of men and women.” [7]. In order to realize these expectations, the MF of LU is undergoing a process which can be described as follows: “It is often observed that the development and the implementation of gender- and diversity-conscious and at the same time discrimination-critical strategies are accompanied by institutional asynchronicities and also individual uncertainties, difficulties or helplessness of teachers.” [20]. The implementation of gender sensitivity into medical studies is an important part in this process. The introduction of the elective in the winter semester 2010/11 was a definite achievement. Yet the work has just begun – an elective for twelve medical students per semester does not make the MF of LU a competence centre for gender-sensitive teaching. The process at the MF of LU has – successfully – just started.

## Acknowledgements

The authors thank the Medical Faculty of Leipzig University (deanship and teaching department) for the continuous support of the elective “career management for medical students” and in the writing of this article.

## Competing interests

The authors declare that they have no competing interests.
